# Reporting of screening and diagnostic AI rarely acknowledges ethical, legal, and social implications: a mass media frame analysis

**DOI:** 10.1186/s12911-020-01353-1

**Published:** 2020-12-10

**Authors:** Emma K. Frost, Stacy M. Carter

**Affiliations:** Australian Centre for Health Engagement, Evidence and Values (ACHEEV), School of Health and Society, Faculty of Arts, Social Sciences, and Humanities, University of Wollongong, Northfields Avenue, Wollongong, NSW 2522 Australia

**Keywords:** Artificial intelligence, Ethics, Frame analysis, Media framing, Screening, Diagnosis

## Abstract

**Background:**

Healthcare is a rapidly expanding area of application for Artificial Intelligence (AI). Although there is considerable excitement about its potential, there are also substantial concerns about the negative impacts of these technologies. Since screening and diagnostic AI tools now have the potential to fundamentally change the healthcare landscape, it is important to understand how these tools are being represented to the public via the media.

**Methods:**

Using a framing theory approach, we analysed how screening and diagnostic AI was represented in the media and the frequency with which media articles addressed the benefits and the ethical, legal, and social implications (ELSIs) of screening and diagnostic AI.

**Results:**

All the media articles coded (n = 136) fit into at least one of three frames: social progress (n = 131), economic development (n = 59), and alternative perspectives (n = 9). Most of the articles were positively framed, with 135 of the articles discussing benefits of screening and diagnostic AI, and only 9 articles discussing the ethical, legal, and social implications.

**Conclusions:**

We found that media reporting of screening and diagnostic AI predominantly framed the technology as a source of social progress and economic development. Screening and diagnostic AI may be represented more positively in the mass media than AI in general. This represents an opportunity for health journalists to provide publics with deeper analysis of the ethical, legal, and social implications of screening and diagnostic AI, and to do so now before these technologies become firmly embedded in everyday healthcare delivery.

## Background

In the broad field of Artificial Intelligence (AI), healthcare is a rapidly expanding area of application [1], enabled by increasing volumes of digital healthcare data, as well as developments in computing power and technologies which allow this new data to be processed [2]. AI tools including machine learning, natural language processing, speech recognition, computer vision, and automated reasoning techniques [2] show promise for application in healthcare contexts [3, 4]. Research into healthcare AI describes significant investment in the potential of these new technologies to change the way healthcare is delivered, and considerable excitement—or even hype—about this potential [5]. The technologies in development and testing stages are diverse in application: using computer vision to interpret medical imaging, using machine learning techniques to identify biomarkers for disease, developing speech recognition technologies so computers can act as counsellors, or creating robots that can perform surgeries autonomously [6]. Emanuel and Wachter [5] describe this optimistic vision of a revolutionised healthcare as the catalyst for substantial venture capital investment.

One area of particular interest for AI application in healthcare is screening and diagnosis, where significant advances have been made in the last half-decade. Machine vision in radiology is especially well-advanced [7], and AI is likely to be integrated into image-based screening programs such as breast screening in the near future [8]. AI development is also relatively mature in cardiology, with machine learning applications being used for example to augment echocardiography reading, to process nuclear cardiography images, or to combine image reading and clinical data to produce diagnostic recommendations [9]. Screening and diagnostic AI augments, or in some cases potentially replaces, clinical skills and practices that have traditionally been central to medical identity and professional responsibility [3]. These technologies also seem likely, in future, to determine or at least influence the pathways of care that open and close to patients. Given these clinically and professionally important roles and impacts, the use of AI for screening and diagnosis is arguably of special significance. In this paper, we present a systematic analysis of media coverage of AI for screening and diagnosis, to understand the ways in which these technologies are being framed, especially for the general public. This analysis was particularly informed by an interest in the ethical, legal, and social implications (ELSIs) of these technologies, an issue to which we now turn.

### The ethical, legal, and social implications of AI in screening and diagnosis

A number of authors have raised questions about the ethical, legal, and social implications of screening and diagnostic AI. For a more detailed description see Carter et al. [3]: interconnected issues of concern include lack of evidence of clinical benefit, potential automation bias and de-skilling in clinicians, professional autonomy and responsibility, data privacy and protection, protection of patient choice and the ability to contest AI-informed decisions, explainability and interpretability of algorithms, and the potential to increase discrimination and bias. The following is a brief discussion of some of these issues.

One commonly identified problem is a deficiency in policy and legislation around AI use [3, 4]. Relatedly, healthcare AI are often developed by commercial entities, but built from public healthcare data, requiring data-sharing arrangements that may not be transparent to or legitimated with the patients who provided those data [4]. Governments have in some instances sold or given large volumes of citizens’ data to private companies [3] with few guarantees regarding the standard to which the resultant algorithms are held [10], or the benefits that may be returned to citizens.

Without a sufficient governance framework, there are further challenges with liability related to AI. Since these tools are effectively augmenting or even replacing decisions previously made by medical professionals, there are questions surrounding who takes responsibility for erroneous outcomes associated with an algorithm’s decisions [4, 6].

Algorithms’ reliance on training data also has implications for data quality. Despite persistent claims to the contrary, algorithms are neither objective nor resistant to bias; values and biases inherent in datasets are not automatically ameliorated by AI analyses [3]. Since algorithms learn from the datasets with which they are trained, implicit biases within that dataset will be reflected in the resultant algorithms [4, 11]. Researchers have argued that algorithms require good and unbiased data to be effective and generalisable [4, 11]. Whilst this is true, it is also important to consider the implicit values encoded into any dataset for results to be interpreted appropriately, and to take seriously the challenges of transferring algorithms between settings [3]. Inasmuch as these ethical, legal, and social implications are significant for citizens who may receive services involving healthcare AI, the way they are discussed in the public domain is important for public understanding.

### Media framing

To better understand this public domain discussion, we conducted a frame analysis of recent media articles reporting on screening and diagnostic AI. Frame analysis was popularized by Entman [12] as a method for analysing the way journalists represent issues in the media. He suggested that ‘frames’ are created when information is knowingly or unknowingly emphasised in, or omitted from, texts. According to Entman, frames construct a reality in four dimensions; they (a) define a problem, (b) diagnose causes, (c) deliver moral judgements, and (d) suggest remedies.

The way AI has been portrayed in the media is something that has been investigated in the past [13], but these efforts have typically focused on examining the impact of negative media portrayals of AI on shaping the public’s fear and disengagement with AI tools [14, 15]. This fear and disengagement with AI has also been the subject of research [16].

Studies investigating media framing of AI, however, have discovered that news coverage of AI is more positive than it is negative [14, 15]. Likewise, a cursory glance at recent news coverage of Healthcare AI reveals a largely optimistic reporting style [17, 18]. As part of a larger project investigating the ethical, legal, and social implications of AI in screening and diagnosis, we were interested in understanding media framing of AI in this context. To our knowledge, this is the first study investigating media framing of any healthcare AI.

Nisbet [19] adapted framing theory to develop a typology of eight frames to categorise science communication in the media. Each frame describes a different way of defining and describing science-related issues. There are eight frames in Nisbet’s typology: Social Progress, Economic Development and Competitiveness, Morality and Ethics, Scientific and Technical Uncertainty, Pandora’s box/Frankenstein’s monster/Runaway science, Public Accountability and Governance, Middle way/Alternative Path, and Conflict and Strategy. A description of these frames can be found below (Table 1). We have utilised this typology in the present study to characterise media framing of Healthcare AI.

**Table 1:** Nisbet's [19] framing typology

Nisbet frame	Nisbet’s description of this frame^a^
Social progress	“A means of improving quality of life or solving problems; alternative interpretation as a way to be in harmony with nature instead of mastering it”
Economic development	“An economic investment; market benefit or risk; or a point of local, national, or global competitiveness”
Conflict and strategy	“A game among elites, such as who is winning or losing the debate; or a battle of personalities or groups (usually a journalist-driven interpretation)”
Morality and Ethics	“A matter of right or wrong; or of respect or disrespect for limits, thresholds, or boundaries”
Scientific and technical uncertainty	“A matter of expert understanding or consensus; a debate over what is known versus unknown; or peer-reviewed, confirmed knowledge versus hype or alarmism”
Pandora’s box/Frankenstein’s monster/runaway science	“A need for precaution or action in face of possible catastrophe and out-of-control consequences; or alternatively as fatalism, where there is no way to avoid the consequences or chosen path”
public accountability and governance	“Research or policy either in the public interest or serving special interests, emphasizing issues of control, transparency, participation, responsiveness, or ownership; or debate over proper use of science and expertise in decision-making (“politicization”)”
Middle way	“A third way between conflicting or polarized views or options”

^a^These frames and descriptions are taken directly from Nisbet [19]

Our overall aim was to explore how screening and diagnosis applications of artificial intelligence in healthcare are framed and explored in the media, with a particular emphasis on the extent to which ethical, legal, and social implications were addressed.

## Methods

We searched media article databases ProQuest and Factiva on 9 April 2020 with the search terms below (Box 1). We collected all newspaper articles, blog posts, magazine articles, press releases, and presentations dated between 1 April 2019 and 31 March 2020 that addressed artificial intelligence applications in screening and diagnosis. All media types were included to allow us to analyse the range of arguments and narratives circulating in the media landscape.

**Box 1:** Terms used for media article search

(" AI " OR "ARTIFICIAL INTELLIGENCE" OR "MACHINE LEARNING") AND ("SCREENING TEST" OR "SCREENING FOR" OR "DIAGNOSIS OF" OR "DIAGNOSING" OR "TEST FOR" OR "TESTING FOR") AND ("HEALTH" OR "HEALTHCARE")	

We used constructed week sampling to select a representative random sample of the articles to analyse. As per Luke and colleagues' [20] recommendations, we constructed six ‘weeks’ to best account for weekly news cycles. We assigned numbers to each week which fell into the allocated timeframe and used a random number generator to select six random instances of each day of the week. As such, 42 days were selected within the timeframe, comprised of 6 random Mondays, 6 random Tuesdays, 6 random Wednesdays, and so on until Sundays. We collected all articles from the database search that fell in these 42 days.

We utilised Nisbet’s [19] framing typology as a deductive framework to code media articles. The frames and their definitions are provided in Table 1. Frames were not mutually exclusive; articles were coded to as many frames as were relevant.

Similar deductive approaches have been recommended in other public health media framing research [21]. Text was coded that specifically addressed one or more of Entman’s four frame features: (a) defining a problem, (b) diagnosing causes, (c) delivering moral judgements, and (d) suggesting remedies. We included additional metrics in the coding framework to collect basic information about the articles’ source publications, source countries, date, health condition(s) being addressed, and commercial information about any AI technologies that were being discussed (technology name, company name). We also collected whether benefits and ELSI were mentioned. Our coding instrument is provided as supplementary material to this article (Additional file 1). 

To determine inter-rater reliability, both authors coded the first 25 articles. Raw agreement in frame allocation came to 89%. Pooled Kappa [22] was 0.66 (95% CI = 0.53–0.79) which indicates a moderate to substantial interrater agreement [23]. Discrepancies were resolved before the remaining articles were coded.

## Results

**Fig. 1:**
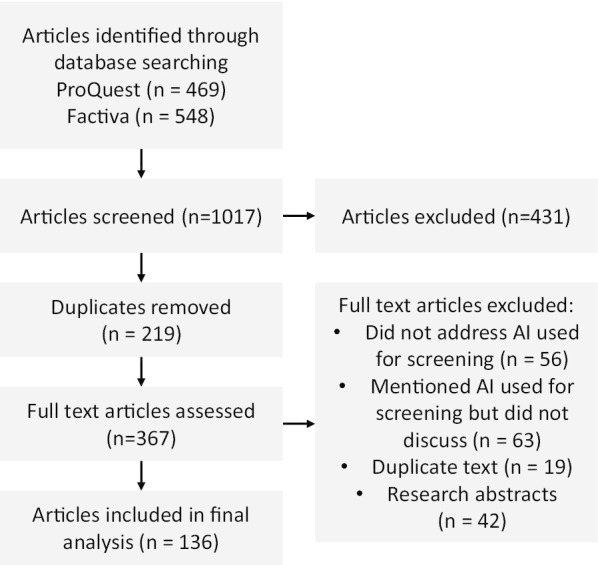
Flow diagram of inclusion process

1017 articles were identified by database searching on the nominated 46 days, of which 431 were initially excluded for irrelevancy (Fig. 1). Of the remaining 586, duplicates were removed (n = 219) and then full texts were reviewed. Of the remaining 367 articles, 56 articles were removed because they did not address the use of AI for screening or diagnosis. A further 63 mentioned AI used for screening or diagnosis in passing but did not discuss it. Nineteen articles were duplicates that were not identified initially due to having different titles or source names. Finally, 43 articles were initially coded but later removed from the data after careful discussion since they were word-for-word reports on research abstracts. The decision to remove them was because they were a different genre: academic paper abstracts rather than media reports.

Of the final sample (n = 136), the majority were articles from various news sources (78.7%; n = 107). The remaining 21.3% was comprised of press releases (n = 18), blog posts (n = 9) and magazine articles (n = 2). Across the week days, Wednesday had the highest count of articles (n = 27; 19.9%), although they were distributed relatively evenly across Monday through Friday, with fewer articles published on Saturdays (n = 6) and Sundays (n = 12).

Whilst some articles addressed multiple health issues or discussed AI in screening and diagnosis more broadly (n = 21), most of the articles addressed one specific health issue (Table 2). Most commonly this was cancer (n = 51; 37.5%), followed by cardiovascular disease (n = 9; 6.6%). Our full dataset is provided as supplementary material to this article (Additional file 2).

**Table 2:** Health conditions addressed in each article

Health condition	Count	% total
Cancers (multiple)	16	11.8
Cardiovascular disease	9	6.6
Colorectal cancer	8	5.9
Breast cancer	7	5.1
Mental health	7	5.1
Alzheimer's disease	6	4.4
Lung cancer	6	4.4
Diabetic retinopathy	5	3.7
Kidney disease	5	3.7
Prostate cancer	4	2.9
Eye conditions	3	2.2
Bowel cancer	2	1.5
COVID-19	2	1.5
Intracranial haemorrhage	2	1.5
Neonatal conditions	2	1.5
Suicide	2	1.5
Various^a^	21	15.4
Other	29	21.3
Total	136	

Articles were coded as ‘various’ if they were not addressing a technology for one specific health condition. E.g. talking about AI in screening in general, or technologies used to screen for a wide range of diseases

The benefits of AI in screening and diagnosis were mentioned in 135 of the 136 articles (99.3%) whilst the ethical, legal, and social implications of the technologies were mentioned in only nine of the articles (6.6%).

### Frame analysis

After coding, we developed a plan for characterising and reporting on frame characteristics, which involved refining Nisbet’s eight frames into three. The Morality and Ethics, Scientific Uncertainty, Pandora’s Box, Public Accountability, and Middle Way frames all frequently co-occurred in a small group of articles which were combined into Frame 3—Alternative Perspectives. Although all these frames were present in the articles, they were typically present as components of an argument rather than fleshed out arguments themselves. For example, an article may mention poor governance and scientific uncertainty together as an argument for a more cautious approach to screening and diagnostic AI. As such, this small group of articles shared a set of common characteristics and arguments. Combining them allowed for analysis of common themes. Similarly, the one instance of the Conflict and Strategy frame co-occurred with the Economic Development frame and shared conceptual traits, so these two Nisbet frames were combined into Frame 2. With a larger sample, it may have been possible to retain more of Nisbet’s original frame structure for analysis.

The Social Progress frame was identified in 96.3% of articles (n = 131) and Economic Development/Conflict and Strategy in 43.4% (n = 59). The Alternative Perspectives frame was found in only 6.6% (n = 9) articles (Table 3).

**Table 3:** Tally of articles in each frame

Frame	Count (%)	Nisbet frame	Count (%)
Frame 1—Social progress	131 (96.3)	Social progress	131 (96.3)
Frame 2—Economic development/conflict and strategy	59 (43.4)	Economic development	59 (43.4)
		Conflict and strategy	1 (0.7)
Frame 3—Alternative perspectives	9 (6.6)	Morality and ethics	4 (2.9)
		Scientific and technical uncertainty	5 (3.7)
		Pandora’s box/Frankenstein’s monster/runaway science	6 (4.4)
		Public accountability and governance	5 (3.7)
		Middle way	3 (2.2)

Descriptions of frames from Nisbet [19]

### Frame 1: Social progress

The social progress frame dominated the rhetoric and was the dominant narrative in most of the articles. Broadly, this frame described a necessity to develop strategies for overcoming diseases and ailments, which represent large burdens on the health system and cause preventable death and disease.

In the social progress frame, diseases were problematized, typically by highlighting that they had an “increasing incidence” [A105] [24] or were “the leading killer in the world” [A82] [25]. Stories in the social progress frame typically implied problems were caused by inefficient current practices in screening and diagnosis which were characterised as “slow” [A21] [26], “subjective” [A10, A27, A195, A244] [27–30], “challenging” [A244] [29] and “manual” [A5] [31]. It was sometimes reinforced that these inefficient practices were overwhelming doctors and impeding their workflow or damaging their ability to spend time engaging with their patients.

With these issues laid as a foundation, the moral judgement implied in the articles in the social progress frame was that AI in screening and diagnosis was a good and important, or at least an inevitable, solution to address disease morbidity and mortality more effectively. In many of these articles, comment was sought from those with a stake in either developing, researching, or implementing the technology. Quotes were selected which reinforced the salience of the technology, emphasising that the technology represented a “pivotal moment in healthcare history” [A42] [32].

At surface level, the suggested remedy was the AI screening or diagnosis technology (or in some cases, technologies) that the article was reporting on. This was clear in the rhetoric which, in contrast to their description of current screening practices, characterised AI screening and diagnosis tools with a different vocabulary. Whilst current practices were slow, AI was quick and simple [A252] [33]; whilst current practices were subjective, AI was “quantitative” [A45] [34] and “objective” [A159, A45] [34, 35].

These technologies were sometimes constructed as being key to a pivotal change in the healthcare system. Sometimes, the importance of quick and easy screening was described in light of a transition within health systems from treatment to prevention [A42] [32], or it was claimed that broader screening will lead to earlier identification of issues and thus better outcomes [A103] [36]. This positioned AI as an important development towards lifting disease burden:

… informed and strategically directed advanced data mining, supervised machine learning, and robust analytics can be **integral, and in fact necessary,** for health care providers to detect and anticipate further progression in this disease. [A88 [37]; emphasis added]

### Frame 2: Economic development/conflict and strategy

The Economic Development frame was the second most common of Nisbet’s frames found in the articles. It overlapped conceptually with the single example of Conflict and Strategy found in the sample and as such, they will be addressed as one for this analysis. All the articles in this frame coincided with instances of the Social Progress frame, so the arguments are not entirely distinct, with this frame tending to borrow from the strength of the Social Progress narrative. However, the Economic Development/Conflict and Strategy (ED/CS) frame tended to focus more dominantly on monetary rather than human costs, and commercial ventures rather than the diversity of projects reported on in the Social Progress frame.

Problem definition and causal attribution were often similar to the Social Progress frame with authors first problematizing the impact of a disease (or multiple diseases), and attributing the problem to slow, subjective, or inefficient current systems. Sometimes, however, articles in the ED/CS frame additionally discussed the monetary cost of that the disease represents (e.g. “*In 2019, … dementias will cost the nation $290 billion”* [A88] [37]).

The moral judgements made in the ED/CS frame were more economically focused than that in the Social Progress frame. These articles generally sought comments from individuals with commercial interests in the technologies being reported on. The worth and value of their commercial endeavours was often associated with their contribution to both social and economic progress, “delivering effective healthcare” [A148] [38] and moving toward “commercialisation” [A98] [39]. Often in these articles, algorithms were described as products which were developed to “disrupt” [A74] [40] a “market” [A83, A137] [41, 42].

The instance of the Conflict and Strategy perspective, in this case, was an extension of these values into venture capitalism where the article described the company responsible for development of the algorithm as aiming to become “one of the top radiogenomics networks in the United States” [A68] [43].

Implicit in this moral assessment was the argument that capitalist ventures such as these were important for social as well as economic progress. As such, the suggested remedy in these articles was again very homogenous, with articles tending to document the technologies developed by one individual company, or one company’s technology, which was the key to reducing the economic costs associated with a disease. Ergo, technologies tended to be represented as economic solutions to largely economic problems.

By offering a method to track progression using only a mobile phone or tablet … the company **aims to stem the cost** of monitoring and screening for Alzheimer’s and related dementias in an aging population. [A18 [44]; emphasis added]

### Frame 3: Alternative perspectives

Each of the Morality, Pandora’s Box, Scientific Uncertainty, Middle Way, and Governance frames from the Nisbet typology were present in some articles. However, they were indistinct from one another as they tended to be present, together, in articles that adopted a more neutral stance compared to those coded to other frames. As such, we dubbed the conglomeration of these frames, ‘Alternative Perspectives’. Nine total articles fit into the alternative perspectives frame, and generally more than one of Nisbet’s 5 initial frames which comprised the alternative perspectives frame were represented in each article (median 2; max 5). This Alternative Perspectives frame overlapped entirely with articles which discussed ELSIs. That is, the nine articles coded into this frame are the same nine which discuss ELSIs of healthcare AI. Table 4 outlines which ELSIs were discussed in the nine articles. Despite being relatively heterogenous within themselves, the articles which fell into Frame 3 were distinct in content and tone from the rest of the sample.

**Table 4:** ELSIs discussed in the nine alternative perspectives articles

Article no. (ref); title	Short description of ELSIs
A143 [50]; Medical AI can now predict survival rates—but it’s not ready to unleash on patients	Historical bias—algorithms that use historical data may produce biased outputs (e.g. algorithms may find a relationship between a disease and a minority group that has historically had worse access to healthcare)
	Black box systems—problems arise when doctors cannot access information about the features algorithms use to produce outputs
	Physician deskilling—doctors may become over-reliant on algorithms to make decisions and lose the skills to make those decisions without the aid of algorithms
A22 [46]; Paging Doctor AI: Artificial intelligence promises all sorts of advances for medicine. And all sorts of concerns	Harm to patients—if AI fails to integrate into workflows or is poorly validated for clinical use it may lead to worse patient outcomes
	Value tension between health and for-profit enterprise—AI is proprietary and there is a value collision with the bedside clinician
	Impact on clinician workflow—AI may be given authority over clinician workflow (e.g. patients’ insurers may only reimburse for the treatments an algorithm recommends, meaning clinicians lose their ability to exercise their own discretion in treating patients)
	Exacerbation of human bias—when algorithms are not designed to take structural inequalities into account, they will produce flawed results
A93 [49]; Genetic Testing Companies Take DNA Tests To A Whole New Level	Concerns about data privacy—using AI tools routinely will raise the need for better data protection regulations
A91 [47]; From suicide prevention to genetic testing, there's a widening disconnect between Silicon Valley health-tech and outside experts who see red flags	Lacking involvement with medical research—concerns developers of AI are not using normal channels for testing and disseminating algorithms. Claims that they make to consumers are unvalidated and the safety of innovations are not regulated
	Poor transparency protocol in tech companies
	Value tension between health and for-profit enterprise—tech emphasises disruption and convenience, whereas healthcare emphasises safety. The values behind AI development conflict with the Hippocratic oath
	Harm to patients—poorly implemented algorithms may lead to iatrogenic health impacts
A3 [45]; The AI governance challenge	Need for better data protection regulations
	Value tension between public and for-profit values
A113 [51]; How A.I. Can Save Your Life	Concerns about data privacy
A117 [52]; How tech giants like Google are targeting the seismic NHS data goldmine	Concerns about data privacy—private companies requesting access to public healthcare data
A8 [53]; Addressing Cyber Security Healthcare and Data Integrity	Concerns about data privacy
A260 [48]; Vietnam: AI for early warning about liver cancer	Inaccuracy of AI techniques

Five of the nine articles also coincided with occurrences of the Social Progress frame. So, in many of these articles the Social Progress narrative was also present and, in some cases, dominant. Often, both diseases and AI technologies were problematised, with the article framed as a discussion of both pros and cons of using these technologies.

Of course, AI applications in sectors like healthcare can yield major social benefits. However, the potential for the mishandling or manipulation of data collected by governments and companies to enable these applications creates risks far greater than those associated with past data-privacy scandals. [A3] [45]

These stories implied that the issues related to AI were caused not by the AI technologies themselves, but by the harmful capitalistic values of those developing AI tools (Morality), the AI field’s lack of involvement with traditional medical research (Scientific Uncertainty), or the poor legislation and regulation surrounding AI that let it develop unbridled (Governance).

the values of AI designers or the purchasing administrators are not necessarily the values of the bedside clinician or patient. Those value collisions and tensions are going to be sites of significant ethical conflict. [A22] [46]

The moral judgement made in these articles was that a more careful approach was needed, to harness the important social developments associated with AI but to simultaneously implement more controls so the issues and value conflicts were better managed. Often, in contrast to the other articles in this sample, these authors would seek out field experts who were not involved with the development of the AI tool(s) in question, giving their argument greater credence through impartiality [A91, A260] [47, 48].

Typically, the solution presented by these articles was for a more regulated and cautious approach to AI in screening and diagnosis. Doctors and those in AI development were implored to be ‘ethical’ [A93] [49] and it was proposed that only ‘explainable’ [A143, A22] [46, 50] or ‘auditable’ [A22] [46] algorithms should be implemented.

## Discussion

Our frame analysis found that media representations of AI in screening and diagnosis were overwhelmingly positive. Benefits were mentioned in all but one article, whilst the ethical, legal, and social implications were much less frequently mentioned in only nine articles. Articles typically fit dominantly within the social progress frame, where AI tools were poised as solutions to constrain rampant disease and morbidity; this was sometimes combined with an economic frame that emphasized financial benefit.

Alternative perspectives existed in a small minority of articles. These articles stood out from the rest–despite their heterogeneity—as they presented a more negative perspective; they included the remaining Nisbet frames. There was nothing that stood out about the sources of these articles, which were similarly diverse to the rest of the sample. The ELSI arguments mentioned in these articles were relatively thin; there were implications, for example, of inappropriately market-driven motivations (and a broader recognition of the potential for value conflict around AI), as well as concern regarding evidence of benefit (beneficence) and the need for explainability (which by implication relates both to the value of transparency and the preservation of professional autonomy and responsibility). However, none of these important issues were dealt with in any depth in this sample.

Like existing research on media framing of AI more broadly [14, 15], we found that media representations of AI in screening and diagnosis were predominantly positive. Our own sample, however, was much more positively framed than that of research looking at media representations of AI more broadly. For example, Chuan and colleagues [14] found that 47.6% of their sample of articles covered at least one type of risk, whilst we found ELSIs were mentioned in only 6.6% of our sample. When compared to the types of risks Chuan and colleagues [14] found in the collected media articles in their study, there are no distinct conceptual differences to the ELSIs in our sample. Similar arguments are present such as privacy, ethics, loss of jobs (replaced in this sample with physician deskilling) and unforeseen risks. Only articles discussing ‘threat to human existence’ were not present in the sample discussing screening and diagnostic AI.

Our results point to an apparent discrepancy between media reporting on AI in screening and diagnosis, and media perspectives on AI more broadly. The same risks are acknowledged, but unlike in general AI reporting they are barely acknowledged, appearing in only a tiny minority of stories; accordingly, health AI stories overwhelmingly emphasise *progress*. Healthcare applications for AI are perhaps more easily spun into narratives which emphasise social and personal benefit, and present less obvious harms to the public than, for example, self-driving cars or autonomous weapons. Indeed, Cave and colleagues [16], who presented a series of positive and negative narratives about the future of AI to research participants, reported that the ‘immortality’ narrative, where AI revolutionises medicine and treatment, was one of only two of eight which elicited more excitement than anxiety from participants. This suggests a general appetite for good news stories about medical AI and may help drive the patterns in reporting that we found.

It is important that these positive narratives do not overshadow meaningful discussion about the ELSIs associated with AI in screening and diagnosis. The optimistic and often economically driven argument for implementation of AI in healthcare is cause for concern if it is allowed to dominate the media and prevent discussion about how to develop more ethical healthcare AI.

### Limitations

This study is somewhat limited by scope, as reflected in the study period, search terms, and news media databases used. It is possible that our constructed sample is not representative of the entire year’s media reporting on screening and diagnostic AI, particularly given the reactive nature of news media to current events. It was beyond the scope of this initial study to consider the reasons behind the positive portrayals of screening and diagnostic AI in the media. In future research, an investigation into the impact of articles’ source countries and the presence or absence of conflicts of interest would provide important context to these results, and a more extensive study may provide useful information about developments in reporting over time.

## Conclusion

Our study was the first to examine media perspectives on AI in screening and diagnosis. Results show that perceptions of screening and diagnostic AI in the media are predominantly positive—far more so than reporting on AI more generally—with most articles emphasising that AI is a source of social progress and economic development. We suggest that healthcare AI may be subject to more positive media framing than AI in general, and that very few articles discussed the ethical, legal, and social implications of AI in screening and diagnosis. This represents an opportunity, especially for specialist health journalists, to provide publics with deeper analysis of the ethical, legal and social implications of screening and diagnostic AI, and to do so now before these technologies become firmly embedded in everyday healthcare delivery.

## Supplementary Information

**Additional file 1.** Coding instrument used for data collection.

**Additional file 2.** Full dataset.

## Data Availability

The dataset supporting the conclusions of this article is included within the article and its additional files.

## Abbreviations

AI: Artificial intelligence
ELSIs: Ethical, legal and social implications
ED/CS: Economic development/conflict and strategy
